# Assessing airborne transmission risks in COVID-19 hospitals by systematically monitoring SARS-CoV-2 in the air

**DOI:** 10.1128/spectrum.01099-23

**Published:** 2023-11-08

**Authors:** Shanglin Li, Jiazhen Guo, Yin Gu, Yan Meng, Ming He, Shangzhi Yang, Ziruo Ge, Guanjun Wang, Yi Yang, Ronghua Jin, Lianhe Lu, Peng Liu

**Affiliations:** 1 Department of Biomedical Engineering, School of Medicine, Tsinghua University, Beijing, China; 2 Changping Laboratory, Beijing, China; 3 Beijing Ditan Hospital, Capital Medical University, Beijing, China; 4 State Key Laboratory of Space Medicine Fundamentals and Application, China Astronaut Research and Training Center, Beijing, China; 5 Beijing Zijing Biotechnology Co., Ltd., Beijing, China; National Taiwan University, Taipei, Taiwan

**Keywords:** SARS-CoV-2, aerosol, nosocomial transmission, airborne transmission, designated COVID-19 hospital

## Abstract

The continuous emergence of highly infectious SARS-CoV-2 variants with the capability of immune evasion demands more effective risk assessment and management approaches for infection prevention and control. Controlling nosocomial transmission is critical in response to the COVID-19 pandemic upon the resumption of day-to-day activities. Here, we systematically analyzed the presence of airborne SARS-CoV-2 in the isolation ward and within the contaminant, emergency, and clean zones of a designated COVID-19 hospital. A total of 359 air samples collected in the clean and emergency zones tested negative for SARS-CoV-2, demonstrating the interruption of airborne transmission in a designated COVID-19 hospital by combining the closed-loop management, unidirectional airflow, and negative pressure of isolation wards. In contrast, some air samples in the corridor outside the isolation wards tested positive for SARS-CoV-2. The leakage of virus-containing air from patient rooms to adjoining areas during rounds and meal delivery sheds light on the need for intensive and widespread virus monitoring in designated COVID-19 hospitals. In conclusion, by systematically monitoring viruses in the air, the study provides a starting point for the standardization of transmission risk management in designated hospitals for the future pandemic of a newly emerged respiratory infectious disease.

**IMPORTANCE:**

Risk management and control of airborne transmission in hospitals is crucial in response to a respiratory virus pandemic. However, the formulation of these infection control measures is often based on epidemiological investigations, which are an indirect way of analyzing the transmission route of viruses. This can lead to careless omissions in infection prevention and control or excessively restrictive measures that increase the burden on healthcare workers. The study provides a starting point for standardizing transmission risk management in designated hospitals by systemically monitoring viruses in the air of typical spaces in COVID-19 hospitals. The negative results of 359 air samples in the clean and emergency zones demonstrated the existing measures to interrupt airborne transmission in a designated COVID-19 hospital. Some positive cases in the corridor of the contaminant zone during rounds and meal delivery highlighted the importance of monitoring airborne viruses for interrupting nosocomial infection.

## INTRODUCTION

The continuous emergence of highly infectious SARS-CoV-2 variants capable of evasion from the vaccine and antibody-mediated immune responses attracted attention worldwide, attaching the great importance of risk assessment and management for preventing coronavirus transmission (1 – 3). Droplet and fomite transmission of SARS-CoV-2 alone could not account for the numerous super-spreading events caused by newly emerged variants (4, 5). The airborne transmission of SARS-CoV-2 via aerosols was now widely recognized (4, 6, 7). Aerosol is a colloid composed of liquid or solid particles suspended in the gas with diameters ranging from a few nanometers to hundreds of micrometers (8, 9). Exhaled droplets ≤10 µM from COVID-19 patients can quickly evaporate into droplet nuclei in dry air within tenths of seconds (9, 10). These fine droplet nuclei may accumulate in still air and spread over long distances, increasing the risk of airborne transmission of SARS-CoV-2 (10). The controversy surrounding the ideal interventions to interrupt or reduce the airborne transmission of SARS-CoV-2 highlights the need for a better understanding of the airborne transmission pathway of this virus (11). Our group has developed an automatic microsystem to precisely monitor the presence of airborne SARS-CoV-2 in public spaces, providing a tool for systematic analysis of the airborne transmission of this coronavirus in various scenarios and spaces (12).

COVID-19 hospitals served as critical sites for the treatment and management of COVID-19 patients, playing a vital role in the response to the pandemic (13, 14). To combat the spread of coronavirus, approximately 1,512 hospitals were constructed across 363 cities in China (15). The infection surveillance and control program of COVID-19 hospitals in China was based on a closed-loop management system that effectively controlled the spread of the virus within hotels and the Olympic competition venues during the 2022 Beijing Winter Olympics (16 – 18). Closed-loop management involves controlled and isolated interactions between patients, staff, commuting routes, epidemic disposal, and logistical support to prevent the spread of COVID-19 within the closed loop, particularly to the outside community (19). After 3 years of rigorous control measures, these COVID-19 hospitals have faced significant financial pressure and endured immense mental and physical stress from their staff (20, 21). In light of these challenges, it is necessary to address them systematically and economically in a scientific manner from the aspect of airborne transmission and monitoring of airborne SARS-CoV-2 as follows.

First, what measures should be implemented to effectively contain the transmission of SARS-CoV-2 from designated COVID-19 hospitals into society? Clear and well-defined measures can strengthen the prevention of the COVID-19 epidemic and alleviate the financial burden on COVID-19 hospitals (22). Conducting a systematic analysis of transmission risks by monitoring airborne SARS-CoV-2 in typical spaces is essential for establishing scientific measures to prevent the coronavirus spread within COVID-19 hospitals.

Second, what standards should be implemented to enhance the protection of healthcare workers and alleviate the physical and mental strain on medical staff? Medical staff protection from coronavirus infection necessitates the use of personal protective equipment (PPE) such as surgical masks, protective suits, safety goggles, gloves, and protective boots (23, 24). By monitoring airborne viruses in high-risk areas like triage wards, patient hospitalization areas, and medical diagnosis zones within COVID-19 hospitals, a scientific understanding of transmission risks can be obtained. Accurate knowledge of transmission risks in various hospital settings is essential for raising awareness of protection measures and alleviating mental stress on healthcare workers (25).

Third, what emergency response plan should be developed to effectively respond to unforeseen pandemics for designated COVID-19 hospitals? Anticipating potential pandemics is essential in safeguarding against overwhelming healthcare systems in the future (26). Implementing airborne virus testing in public spaces will enhance the capacity of COVID-19 hospitals by enabling the simultaneous testing, isolation, and discharge of a group of individuals.

Finally, what measures should be implemented for rapidly and flexibly transitioning from a pandemic emergency to routine servicing? Each wave of the COVID-19 epidemic in China has been caused by new coronavirus variants and increased financial strain on designated COVID-19 hospitals (20). Enabling a rapid recovery of routine services is crucial in reducing the financial burden on hospitals (27). Monitoring airborne viruses in hospital settings can help assess transmission risks and prevent cross-infection during the reopening process, ultimately contributing to a smooth transition from pandemic emergency to routine servicing.

Utilizing an ultrasensitive airborne SARS-CoV-2 detection system, we conducted a systematic monitoring of airborne SARS-CoV-2 in typical spaces within contaminant, emergency, and clean zones. From the perspective of risk assessment and management in designated COVID-19 hospitals, we explored potential solutions to these four challenges based on the results of our airborne virus testing. In conclusion, this study provides a theoretical foundation for standardizing SARS-CoV-2 transmission risk assessment and risk management practices in COVID-19 hospitals by utilizing the newly developed tool for monitoring airborne viruses.

## MATERIALS AND METHODS

### Monitoring airborne SARS-CoV-2 in the designated COVID-19 hospital

**Fig 1:**
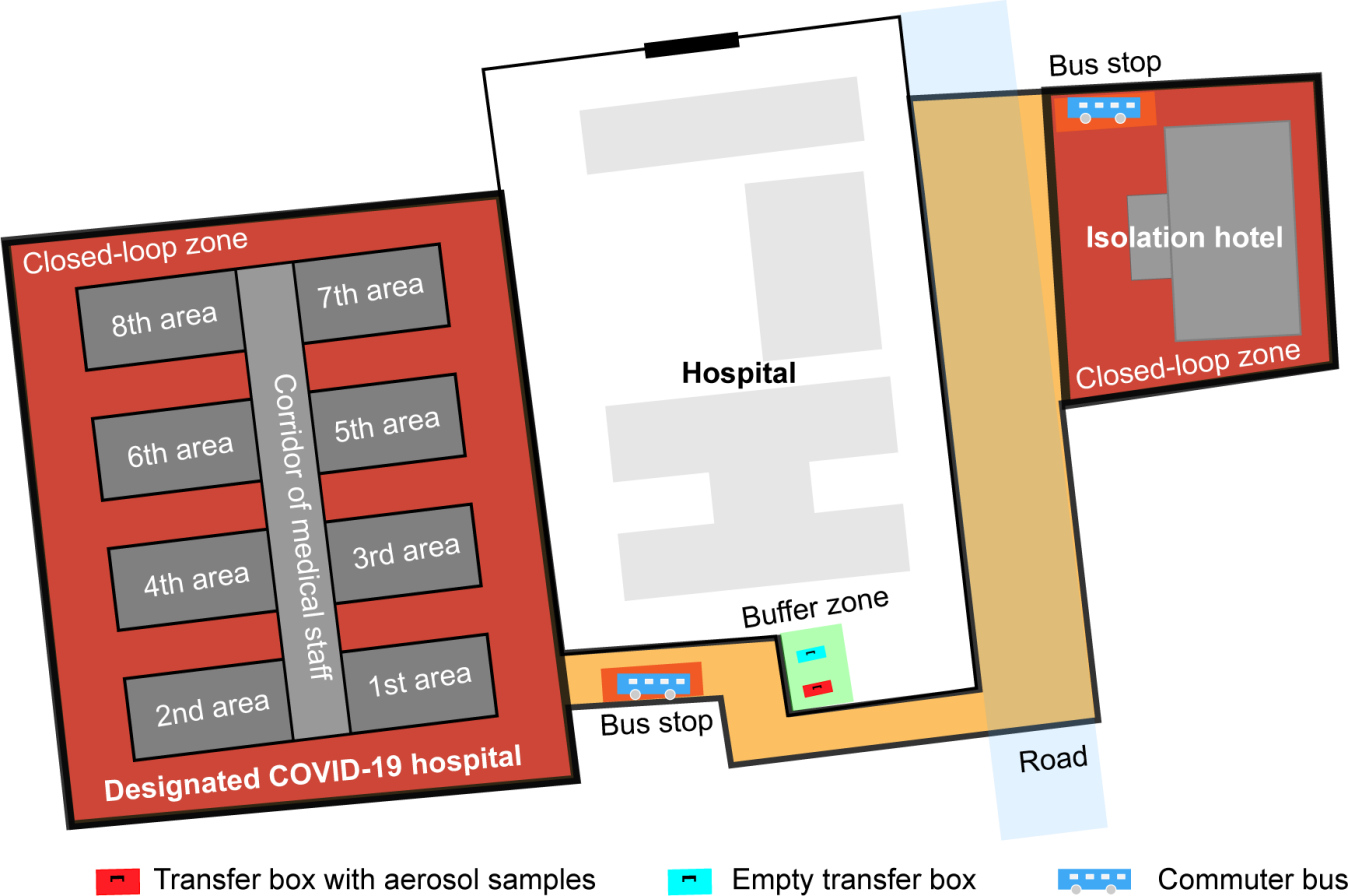
The closed-loop management of the designated COVID-19 hospital and the transport of air samples. The red region is the closed-loop zone. The green region is the buffer zone. The orange zone is the closed-loop route for the commuter bus.

In the designated COVID-19 hospital, the staff were restricted to the closed-loop zone shown in Fig. 1. A buffer zone, the single access corridor, allows for the ingress and egress of staff and supplies. The contaminant, emergency, and clean zones were hierarchically designed for closed-loop management. The contaminant zone was a high-risk space and was located near the isolation ward. The emergency zone was adjacent to the contaminant zone and had a single entrance into the contaminant zone and an exit out of the contaminant zone through the first and second rooms for taking off PPE. The clean zone was only accessible to medical staff and was lateral to the emergency zone.

**Fig 2:**
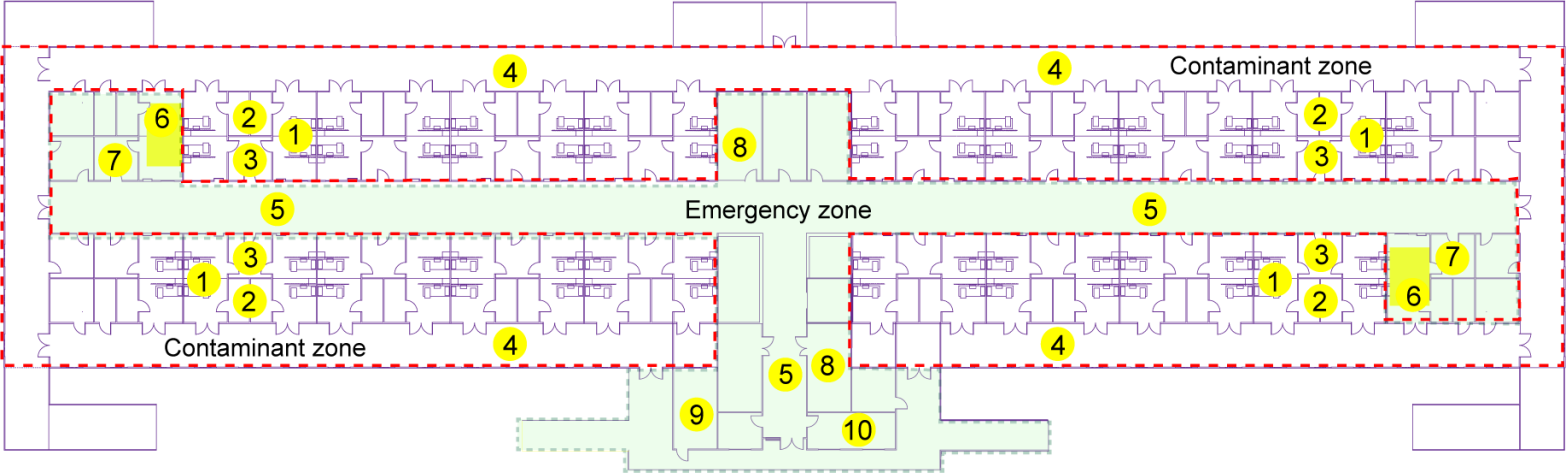
Typical spaces in the designated COVID-19 hospital. 1, the isolation ward. 2, the washroom. 3, the buffer room. 4, the corridor of the contaminant zone. 5, the corridor of the emergency zone. 6, the first undressing room. 7, the second undressing room. 8, the changing room. 9, the electricity distribution room. 10, the storage room. The green zone is the emergency zone for healthcare workers. The area surrounded by the red dotted line is the contaminant zone.

To systematically analyze the airborne transmission of SARS-CoV-2 in the designated COVID-19 hospital under closed-loop management, aerosols and large airborne particles in the isolation ward, contaminant, emergency, and clean zones were collected and analyzed for 27 days. The closed-loop management encompassed the closed-loop of the contaminant, emergency, and clean zones, allowing only the one-way flow of people and materials from the clean zone to the contaminant zone. The designated COVID-19 hospital possessed a functional ventilation system, and the air was uniflow from clean and emergency zones to the contaminant zone. To identify breaches in the current infection control and prevention practices, air samples collected in the emergency and clean zones were systematically collected and analyzed from 14 May to 9 April. The sampling sites in the emergence zone included the corridor of the emergency zone, the first and second undressing rooms, the changing room, the electricity distribution room, and the warehouse on the first floor shown in Fig. 2. The sampling sites in the clean zone included the doctor’s office, lounge, washroom, infection division’s storage room, infection division office, radiology lounge, and lounge for cleaners in the periphery of the emergency zone. The sampling sites covered most of the spaces accessible to medical staff. For some typical spaces such as the corridor of the contaminant zone, doctor’s office, first and second undressing rooms, and washrooms, the sampler was allowed to run for several days, and the sampling frequency was one sample at a sampling site per day. The number of repeated samples corresponded to the human flow and logistics. The detailed information on sampling sites and results are listed in Tables S1 and S2. To assay the airborne spread of SARS-CoV-2 in the contamination zone, air samples in the corridors outside the isolation ward were analyzed during the ward rounds and meal delivery. Similarly, airborne SARS-CoV-2 in the isolation ward was analyzed for safety assessment after discharging the COVID-19 patients. All isolation wards were normally functioning under negative pressure.

### Collection, transportation, and analysis of air samples

During the closed-loop management, air samples were collected from spaces in the closed-loop zone of Beijing Ditan Hospital of the Capital Medical University using a portable cyclone sampler purchased from Beijing Gongjiang Biotechnology (Beijing, China). The portable cyclone sampler shown in Fig. S1 possessing a high flow rate and high collection efficiency was suitable for airborne particles collection in the semi-enclosed space with low gas flow. The height of the cyclone sampler was set at 1.5 m, approximately the height of the human respiratory zone. After positioning the cyclone sampler at the specified position, a 5-mL tube with 3 mL of 1 × lysis buffer (Beijing Zijing Biotechnology Co., Ltd., China) was installed in the bottom of the sampling bucket for collection.

Specimen collection was performed by the sample collection personnel who delivered the collected specimens in a transport box to the buffer zone and back to the closed-loop zone. The sample transport personnel then decontaminated the transport box and its surroundings before taking it out of the buffer zone to the testing center. Entry for sample collection personnel, sampling supplies, and exit of air samples occurred through the designated COVID-19 hospital’s buffer zone (Fig. 1).

Air samples were collected using a portable cyclone sampler (Fig. S1). These samples were analyzed in a biological safety level 3 laboratory using the PowerFlex FeiKe bioaerosol SARS-CoV-2 surveillance system. This system includes a fully automatic detection instrument and a FeiKe aerosol SARS-CoV-2 detection kit purchased from Beijing Zijing Biotechnology Co., Ltd., which can detect as few as 20 copies of the N gene of SARS-CoV-2 RNA in air samples (12).

Simultaneously, air samples were rechecked using a viral RNA extraction and *in situ* amplification method based on a polyacrylamide derivative-modified filter disc (PAD-FD) that can detect as low as 10 copies/test (28). The workflow of the method was as follows: up to 0.5 mL of the collected air samples was transferred into the spin column of the PAD-FD device. The viral RNA was then captured on the PAD-FD via centrifugation at 3,000 g for 1 min in a mini centrifuge. After two washes with 0.5 mL of diethylpyrocarbonate (DEPC) treated water, the supporting mount with the PAD-FD and base was manually unscrewed from the column and covered to a 0.2-mL PCR tube upside down immediately. Gentle tapping allows the filter disc to fall into the tube. The qPCR reaction mixtures were prepared by mixing 2 × One Step PrimeScript III RT-qPCR Mix (Takara, Shiga, Japan) with the primers and probes. All primers and probes were synthesized and purified using high performance liquid chromatography (HPLC) by Sangon Biotech (Shanghai, China). The sequences of primers and probes are listed in Table S1. After transferring the PAD-FD along with captured viral RNA into a PCR tube with 20 µL of the reaction mixture, thermal cycling and fluorescent detection were carried out on an Applied Biosystems 7500 Real-Time PCR System (Thermo Fisher Scientific, Foster City, CA, USA). The RT-qPCR thermal program began at 52°C for 5 min for reverse transcription of viral RNA, 95°C for 30 s to activate the DNA polymerase, and followed by 45 cycles of 95°C for 5 s and 60°C for 30 s. To ensure the accuracy of results, each detection contained four negative controls and two positive controls. Since the cycle threshold (Ct) value of this *in situ* amplification method was always delayed, the result of air samples was considered positive when the Ct value of the target gene was ≤45 and negative when both were >45.

### Collection and analysis of swab samples

The collection and analysis of nasopharyngeal swabs and air samples were approved by the institutional review board of Beijing Ditan Hospital of the Capital Medical University. To analyze the leakage of airborne viruses, nasopharyngeal swabs were collected from COVID-19 patients in corresponding wards with informed consent and analyzed by reverse transcription-PCR (RT-PCR). RT-PCR was conducted with primers and probes targeting the ORF1ab and N genes and a positive reference gene. The reaction system and amplification conditions were performed according to the manufacturer’s specifications (Guangzhou Daan Gene Co. Ltd., China) with registration certificate no. 20203400063. The result was considered valid only when the Ct value of the reference gene was ≤38. The result was considered positive when the Ct values of both target genes were ≤38 and negative when they were both >38. If only one of the target genes had a Ct value ≤38 and the other >38, it was interpreted as a single-gene positive.

## RESULTS

### Monitoring of airborne SARS-CoV-2 in the clean and emergency zones

Most epidemic prevention measures were based on epidemiological investigations, which provided indirect evidence of virus transmission without a scientific basis (21, 29, 30). Monitoring airborne SARS-CoV-2 in public spaces offers direct transmission routes for epidemic prevention and enables the systematic surveillance of transmission risks in COVID-19 hospitals (12). Based on the flow and aggregation of healthcare workers, air samples were systematically collected and analyzed from typical spaces such as the doctor’s office, washroom, lounge, changing room, infection division’s storage room, infection division office, electricity distribution room, the lounge for cleaners, the corridor of the emergency zone, first undressing room, and second undressing room for 20 days. These sampling sites covered major spaces in the emergency and clean zones shown in Fig. 2. The statistical data in Table 1 and detailed results in Table S2 showed that a total of 359 air samples collected in the emergence and clean zones tested negative for SARS-CoV-2 after 20 days of monitoring. By conducting airborne SARS-CoV-2 monitoring in the designated COVID-19 hospital, the feasibility of this activity as a supplementary risk surveillance measure for public spaces was practically verified.

**TABLE 1:** Test results of air samples collected in the emergency and clean zones

Sampling site	Sampling duration (minutes)	Cumulative detection (days)	Detection results (positive/negative case)
Corridor of emergency zone	30	4	0/39
Second undressing room	30	6	0/48
First undressing room	30	12	0/64
Doctor’s office	30	5	0/44
Changing room	30	5	0/45
Lounge	30	7	0/60
Washroom	30	5	0/20
Infection division’s storage room	30	5	0/5
Infection division office	30	5	0/5
Radiology lounge	30	5	0/5
Electricity distribution room	30	5	0/19
Lounge for cleaners	30	5	0/5

### Characterizing airborne transmission of SARS-CoV-2 in the contaminant zone

**Fig 3:**
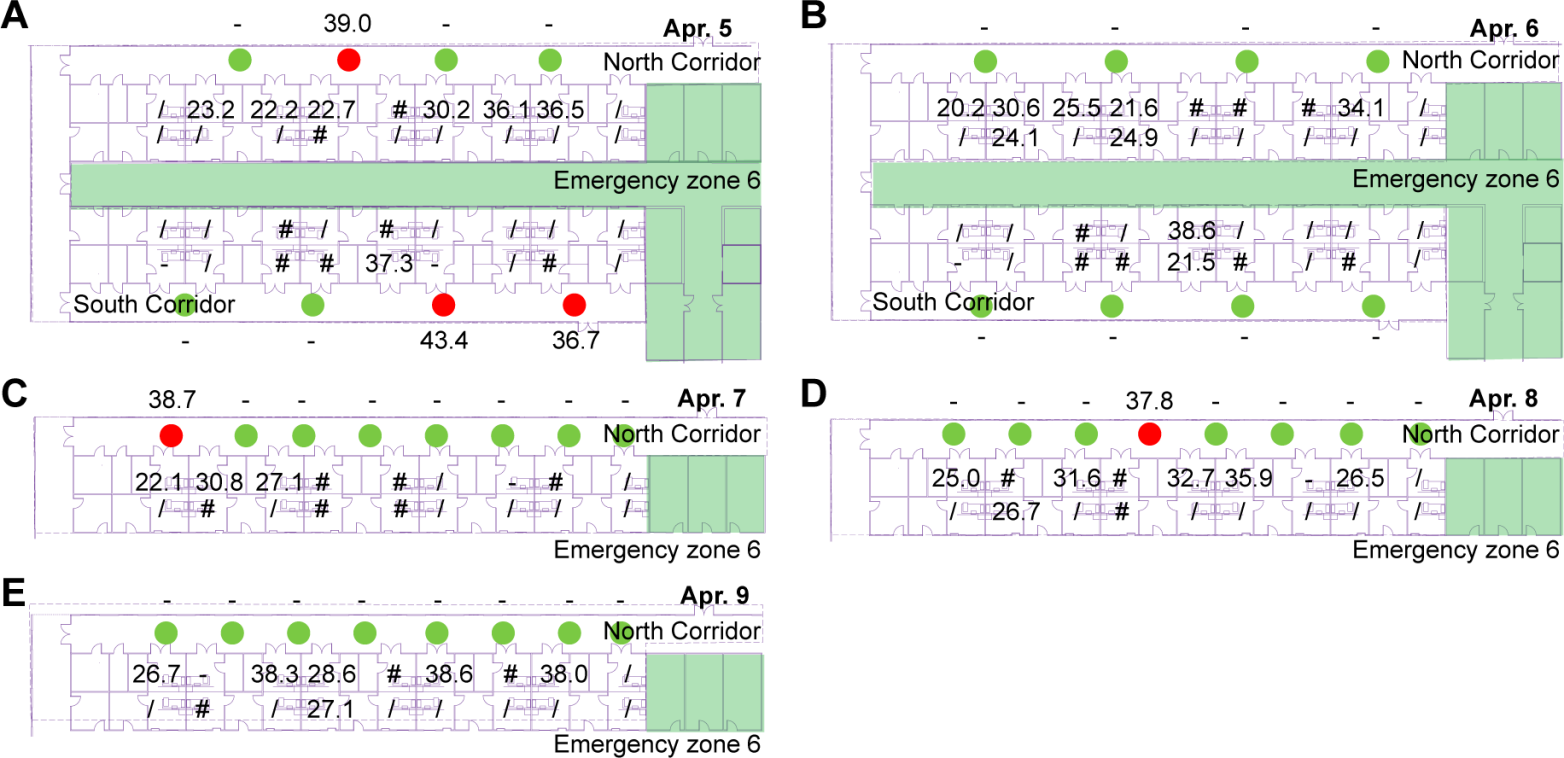
Collection and detection of aerosols in the corridor outside isolation wards. (A) Test results of air samples collected when the doors opened continually during ward rounds. (B) Test results of air samples gathered when doors were closed. (C) Test results of air samples collected when the doors opened occasionally during ward rounds. (D) and (E) Air samples gathered during the meal delivery. Green cycle, the sampling site was negative. Numbers were Ct values of air samples collected in corridors and nasopharyngeal swab samples collected from COVID-19 patients in corresponding isolation wards. The green zone is the emergency zone for healthcare workers. The red circles are the positive sampling sites. The green circles are the negative sampling sites. # means the COVID-19 patients in the ward are without the data of nucleic acid testing. / means the isolation wards are without COVID-19 patients. - means the COVID-19 patients in the ward were tested negative for SARS-CoV-2.

Hospital-acquired COVID-19 infections were directly associated with excess morbidity and mortality in patients and healthcare workers (31, 32). Transmission within hospitals has been identified as a critical concern in response to the COVID-19 pandemic (33, 34). To better characterize the risk of transmission and design more effective infection-control interventions, the transmission route of viral aerosols was analyzed by monitoring airborne viruses in the contaminant zone. As shown in Fig. 3; Table S3, four samples collected during the ward round in the corridor of the contaminant zone tested positive for SARS-CoV-2, while a total of eight air samples gathered when the doors were closed tested negative, indicating the leakage of airborne coronaviruses from wards into the corridor of the contaminant zone through the doors of isolation wards. Furthermore, aerosols and droplets in the corridor were also collected and tested during meal deliveries. One air sample gathered at the corridor tested positive for SARS-CoV-2 on 6 April as shown in Fig. 3, illustrating the spillover of air after opening the doors during meal deliveries. The results showed that even under negative pressure ventilation, the airborne SARS-CoV-2 could still leak out of isolation wards when the door was opened during physicians’ daily rounds and meal deliveries, highlighting the limited effectiveness of negative pressure ventilation and unidirectional flow ventilation in interrupting airborne transmission of COVID-19. The leakage of airborne viruses from the isolation ward also highlighted the necessity of monitoring airborne viruses in designated hospitals to prevent nosocomial infection, verify the effectiveness of the air disinfection system, and uncover errors and omissions in infection prevention and control. Additionally, these results emphasized the need for paying more attention to the airflow of open spaces in designated hospitals, in addition to people and material flow.

### Detection of airborne SARS-CoV-2 in the isolation ward

As physicians performed their daily rounds in the COVID-19 wards, they had direct contact with COVID-19 patients and were exposed to airborne viruses, posing a significant hazard for healthcare workers and their families (35). To assess the transmission risks in the isolation ward, aerosol samples were collected and analyzed. As shown in Table 2, air samples from isolation wards 5, 10, and 16, occupied by COVID-19 patients with high Ct values (>25), all tested negative. In contrast, an air sample collected in isolation ward 4, with a patient having a Ct value of 23.6, and another sample from isolation ward 8, with a patient having a Ct value of 22.4, tested positive, indicating higher risk transmission areas with patients having lower Ct values (Table 2; Fig. S4). However, as these COVID-19 patients recovered, the air samples in isolation wards 4 and 8 became negative, demonstrating an association between viral particle concentration and patient viral load (Table 2). Based on these findings, it was necessary to heighten awareness of infection control processes and reduce physicians’ rounds when patients in the ward with low Ct values (<25) were present. Since severe COVID-19 patients frequently showed higher viral loads, the development of remote patient monitoring technology would be valuable in ensuring the safety of healthcare workers and maintaining the orderly operation of the health service system during future pandemics (36, 37). Additionally, the practical implementation of a fully automatic aerosol-monitoring system would eliminate the need for medical staff to collect samples in contaminated zones and provide real-time reminders of monitoring results for airborne viruses, enhancing protection for healthcare workers.

**TABLE 2:** Test results of air samples collected in the isolation wards^*a*^

Sampling site	Sampling duration (minutes)	COVID-19 patients (Ct value)	Date	Detection results (Ct value)
Empty ward 1	30	Discharged	2 April	Negative
Empty ward 2	30	Discharged	2 April	Negative
Empty ward 3	30	Discharged	2 April	Negative
Empty ward 4	30	Discharged	2 April	Negative
Empty ward 1	30	Discharged	3 April	Negative
Empty ward 2	30	Discharged	3 April	Negative
Empty ward 3	30	Discharged	3 April	Negative
Empty ward 4	30	Discharged	3 April	Negative
Empty ward 5	30	Discharged	3 April	Negative
Empty ward 6	30	Discharged	3 April	Negative
Isolation ward 5	15	Ct 38	4 April	Negative
Isolation ward 10	15	Ct 36	4 April	Negative
Isolation ward 16	15	Ct 26	4 April	Negative
Isolation ward 4	15	Ct 23.6	5 April	Ct 37.5
Isolation ward 8	15	Ct 22.4	5 April	Ct 38.1
Isolation ward 16	15	/	5 April	Negative
Isolation ward 4	15	/	6 April	Ct 41.2
Isolation ward 8	15	/	6 April	Negative
Isolation ward 4	15	/	7 April	Negative
Isolation ward 8	15	/	7 April	Negative

^*a*^ Ct value is a threshold value of PCR related to the viral load of corresponding samples. Discharged means the COVID-19 patient in the empty ward was discharged. / means without the data of nucleic acid testing of the nasopharyngeal swab sample on that day.

After 3 years of recurring COVID-19 outbreaks, COVID-19 hospitals faced significant financial burdens (38, 39). Rapid and flexible resumption of routine services after each pandemic wave was crucial to alleviate the financial strain (40), but ensuring cross-infection prevention was essential for safely resuming routine services. Cross-infection often occurs due to contact between COVID-19 patients and the contaminated environment (41). To prevent cross-contamination during the hospitalization of COVID-19 patients, airborne virus detection in empty isolation wards before admission was necessary. The samples collected from these wards after releasing recovered COVID-19 patients were analyzed, as shown in Table 2. All the results were negative, indicating a successful disinfection process in this designated COVID-19 hospital. This practical test demonstrated the feasibility of the virus monitoring system for identifying risk factors in sterilized wards. To minimize the risk of cross-infection and efficiently utilize isolation wards, especially during unexpected pandemics and the reopening of routine services, it is necessary to monitor airborne viruses in isolation wards.

## DISCUSSION

Airborne transmission of SARS-CoV-2, particularly the latest variants, poses challenges for infection prevention and control in COVID-19 hospitals (4, 42). Incorporating new technologies was crucial for solving these issues that emerged in COVID-19 hospitals. Herein, based on the ultrasensitive SARS-CoV-2 monitoring system developed by Peng Liu’s team at Tsinghua University (12, 28), the airborne spread of SARS-CoV-2 through public spaces in a designated COVID-19 hospital was systematically analyzed by monitoring viruses in the air of typical settings. The negative results of air samples collected in the clean and emergency zones demonstrated the existing measures, including closed-loop management, unidirectional airflow, and negative-pressure wards, to interrupt virus transmission in designated COVID-19 hospitals. The leakage of viral aerosols from isolation wards into corridors even with negative pressure ventilation highlighted the need for periodic monitoring of SARS-CoV-2 aerosols in designated COVID-19 hospitals to identify errors and omissions in infection control and prevention processes. Additionally, monitoring SARS-CoV-2 in the air would enhance the safety of healthcare workers in hospital settings. In conclusion, our study provided a foundation for standardizing infection prevention and control in designated hospitals by utilizing the newly developed tool for monitoring viruses in the air and underscored the need for extensive and comprehensive monitoring of airborne respiratory viruses in these settings.

## SUPPLEMENTAL MATERIAL

Supplemental file 1 spectrum.01099-23-s0001.pdf Fig. S1 and Tables S1 to S4.

ASM does not own the copyrights to Supplemental Material that may be linked to, or accessed through, an article. The authors have granted ASM a non-exclusive, world-wide license to publish the Supplemental Material files. Please contact the corresponding author directly for reuse.
