# Supplementary material for: Assessing airborne transmission risks in COVID-19 hospitals by systematically monitoring SARS-CoV-2 in the air
Source: Microbiol Spectr. 2023 Nov 8;11(6):e01099-23. doi: 10.1128/spectrum.01099-23 (PMC10714815; doi:10.1128/spectrum.01099-23)
Supplement: Supplemental file 1 — Fig. S1 and Tables S1 to S4. [file spectrum.01099-23-s0001.pdf]

Supplementary information for

Assessing airborne transmission risks in COVID-19 hospitals by systematically monitoring SARS-CoV-2 in the air

Shanglin Li[1,3], Jiazhen Guo[2], Yin Gu[5], Yan Meng[2], Ming He[2], Shangzhi Yang[4], Ziruo Ge[2], Guanjun Wang[4], Yi Yang[4], Ronghua Jin[2], Lianhe Lu[2,*], Peng Liu[1,3,*]

1 Department of Biomedical Engineering, School of Medicine, Tsinghua University, Beijing, 100084, China.

2 Beijing Ditan Hospital, Capital Medical University, Beijing, 100015, China.

3 Changping Laboratory, Beijing, 102206, China.

4 Beijing Zijing Biotechnology Co., Ltd., 102206, China.

5 State Key Laboratory of Space Medicine Fundamentals and Application, China Astronaut Research and Training Center, Beijing,100094, China

* Address correspondence to Peng Liu, pliu@tsinghua.edu.cn; Lianhe Lu, looklu@126.com.

First Author and Second Author contributed equally to this work.

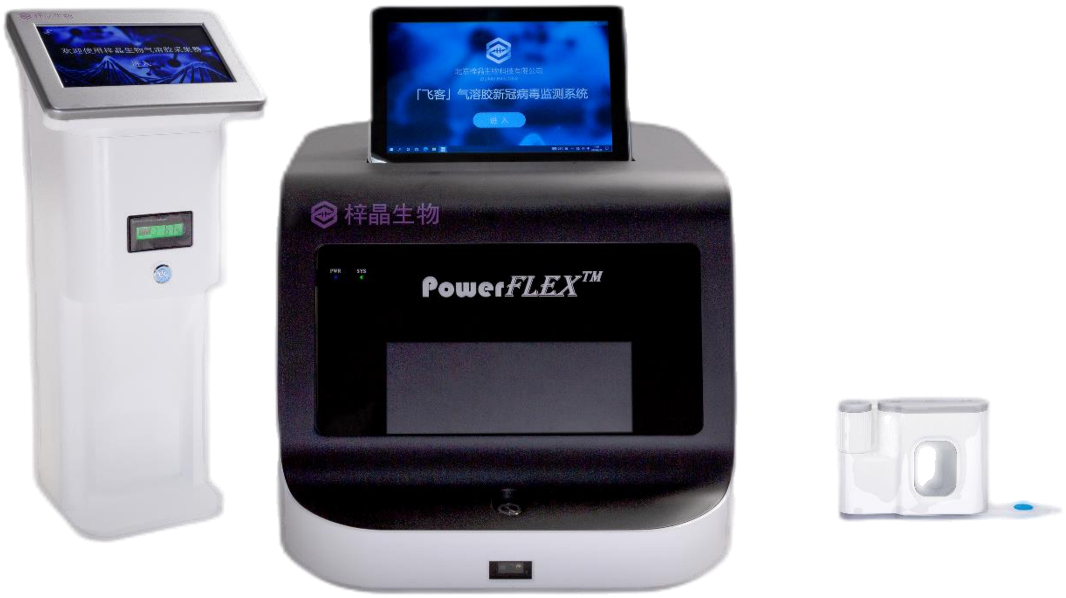

FIG S1. The PowerFLEX™ FeiKe™ Bioaerosol SARS-CoV-2 Surveillance System. The portable cyclone sampler (Left), the fully automatic instrument (Middle), and the detection cassette (Right) used in this work were purchased from Beijing Zijing Biotechnology.

TABLE S1. Sequences and final concentrations of primers and probes

Names	Sequences (5' → 3')	Final conc.
nCoV-N_F	TTA CAA ACA TTG GCC GCA AA	500 nM
nCoV-N_R	GCG CGA CAT TCC GAA GAA	500 nM
nCoV-N_Pr	/FAM/ ACA ATT TGC CCC CAG CGC TTC AG /BHQ1/	125 nM

TABLE S2. Sampling sites and test results of viral aerosols in emergence and clean zones

Sampling date	Sampling point location	Zone	Results	Ct value
2022/3/14	Inside corridor 1	Emergency zone	-	
2022/3/14	Inside corridor 2	Emergency zone	-	
2022/3/14	Inside corridor 3	Emergency zone	-	
2022/3/14	Inside corridor 4	Emergency zone	-	
2022/3/14	Inside corridor 5	Emergency zone	-	
2022/3/14	Inside corridor 6	Emergency zone	-	
2022/3/14	Inside corridor 7	Emergency zone	-	
2022/3/15	Inside corridor 1	Emergency zone	-	
2022/3/15	Inside corridor 2	Emergency zone	-	
2022/3/15	Inside corridor 3	Emergency zone	-	
2022/3/15	Inside corridor 4	Emergency zone	-	
2022/3/15	Inside corridor 5	Emergency zone	-	
2022/3/15	Inside corridor 6	Emergency zone	-	
2022/3/15	Inside corridor 7	Emergency zone	-	
2022/3/15	Inside corridor 8	Emergency zone	-	
2022/3/15	Changing room 1, second floor	Clean zone	-	
2022/3/15	Changing room 2, second floor	Clean zone	-	
2022/3/15	Changing room 3, second floor	Clean zone	-	
2022/3/15	Changing room 4, second floor	Clean zone	-	
2022/3/15	Changing room 5, second floor	Clean zone	-	
2022/3/15	Changing room 6, second floor	Clean zone	-	
2022/3/15	Changing room 7, second floor	Clean zone	-	
2022/3/15	Changing room 8, second floor	Clean zone	-	
2022/3/15	Changing room 9, second floor	Clean zone	-	
2022/3/15	Infection division office	Clean zone	-	
2022/3/15	Storehouse of infection division	Clean zone	-	
2022/3/15	Radiology lounge	Clean zone	-	
2022/3/16	Changing room 1, second floor	Clean zone	-	
2022/3/16	Changing room 2, second floor	Clean zone	-	
2022/3/16	Changing room 3, second floor	Clean zone	-	
2022/3/16	Changing room 4, second floor	Clean zone	-	
2022/3/16	Changing room 5, second floor	Clean zone	-	
2022/3/16	Changing room 6, second floor	Clean zone	-	
2022/3/16	Changing room 7, second floor	Clean zone	-	
2022/3/16	Changing room 8, second floor	Clean zone	-	
2022/3/16	Infection division office	Clean zone	-	
2022/3/16	Storehouse of infection division	Clean zone	-	
2022/3/16	Radiology lounge	Clean zone	-	
2022/3/17	Inside corridor 1	Emergency zone	-	

2022/3/17	Inside corridor 2	Emergency zone	-	
2022/3/17	Inside corridor 3	Emergency zone	-	
2022/3/17	Inside corridor 4	Emergency zone	-	
2022/3/17	Inside corridor 5	Emergency zone	-	
2022/3/17	Inside corridor 6	Emergency zone	-	
2022/3/17	Inside corridor 7	Emergency zone	-	
2022/3/17	Inside corridor 8	Emergency zone	-	
2022/3/17	Changing room 1, second floor	Clean zone	-	
2022/3/17	Changing room 2, second floor	Clean zone	-	
2022/3/17	Changing room 3, second floor	Clean zone	-	
2022/3/17	Changing room 4, second floor	Clean zone	-	
2022/3/17	Changing room 5, second floor	Clean zone	-	
2022/3/17	Changing room 6, second floor	Clean zone	-	
2022/3/17	Changing room 7, second floor	Clean zone	-	
2022/3/17	Changing room 8, second floor	Clean zone	-	
2022/3/17	Changing room 9, second floor	Clean zone	-	
2022/3/17	Changing room 10, second floor	Clean zone	-	
2022/3/17	Infection division office	Clean zone	-	
2022/3/17	Storehouse of infection division	Clean zone	-	
2022/3/17	Radiology lounge	Clean zone	-	
2022/3/18	Inside corridor 1	Emergency zone	-	
2022/3/18	Inside corridor 2	Emergency zone	-	
2022/3/18	Inside corridor 3	Emergency zone	-	
2022/3/18	Inside corridor 4	Emergency zone	-	
2022/3/18	Inside corridor 5	Emergency zone	-	
2022/3/18	Inside corridor 6	Emergency zone	-	
2022/3/18	Inside corridor 7	Emergency zone	-	
2022/3/18	Inside corridor 8	Emergency zone	-	
2022/3/18	Changing room 1, second floor	Clean zone	-	
2022/3/18	Changing room 2, second floor	Clean zone	-	
2022/3/18	Changing room 3, second floor	Clean zone	-	
2022/3/18	Changing room 4, second floor	Clean zone	-	
2022/3/18	Changing room 5, second floor	Clean zone	-	
2022/3/18	Changing room 6, second floor	Clean zone	-	
2022/3/18	Changing room 7, second floor	Clean zone	-	
2022/3/18	Changing room 8, second floor	Clean zone	-	
2022/3/18	Changing room 9, second floor	Clean zone	-	
2022/3/18	Changing room 10, second floor	Clean zone	-	
2022/3/18	Infection division office	Clean zone	-	
2022/3/18	Storehouse of infection division	Clean zone	-	
2022/3/18	Radiology lounge	Clean zone	-	
2022/3/19	Inside corridor 1	Emergency zone	-	
2022/3/19	Inside corridor 2	Emergency zone	-	

2022/3/19	Inside corridor 3	Emergency zone	-	
2022/3/19	Inside corridor 4	Emergency zone	-	
2022/3/19	Inside corridor 5	Emergency zone	-	
2022/3/19	Inside corridor 6	Emergency zone	-	
2022/3/19	Inside corridor 7	Emergency zone	-	
2022/3/19	Inside corridor 8	Emergency zone	-	
2022/3/19	Changing room 1, second floor	Clean zone	-	
2022/3/19	Changing room 2, second floor	Clean zone	-	
2022/3/19	Changing room 3, second floor	Clean zone	-	
2022/3/19	Changing room 4, second floor	Clean zone	-	
2022/3/19	Changing room 5, second floor	Clean zone	-	
2022/3/19	Changing room 6, second floor	Clean zone	-	
2022/3/19	Changing room 7, second floor	Clean zone	-	
2022/3/19	Changing room 8, second floor	Clean zone	-	
2022/3/19	Changing room 9, second floor	Clean zone	-	
2022/3/19	Changing room 10, second floor	Clean zone	-	
2022/3/19	Infection division office	Clean zone	-	
2022/3/19	Storehouse of infection division	Clean zone	-	
2022/3/19	Radiology lounge	Clean zone	-	
2022/3/20	Second undressing room 1	Emergency zone	-	
2022/3/20	Second undressing room 2	Emergency zone	-	
2022/3/20	Second undressing room 3	Emergency zone	-	
2022/3/20	Second undressing room 4	Emergency zone	-	
2022/3/20	Second undressing room 5	Emergency zone	-	
2022/3/20	Second undressing room 6	Emergency zone	-	
2022/3/20	Second undressing room 7	Emergency zone	-	
2022/3/20	Second undressing room 8	Emergency zone	-	
2022/3/20	Doctor's office 1, second floor	Clean zone	-	
2022/3/20	Doctor's office 2, second floor	Clean zone	-	
2022/3/20	Doctor's office 3, second floor	Clean zone	-	
2022/3/20	Doctor's office 4, second floor	Clean zone	-	
2022/3/20	Doctor's office 5, second floor	Clean zone	-	
2022/3/20	Doctor's office 6, second floor	Clean zone	-	
2022/3/20	Doctor's office 7, second floor	Clean zone	-	
2022/3/20	Doctor's office 8, second floor	Clean zone	-	
2022/3/20	Washroom 1, second floor	Clean zone	-	
2022/3/20	Washroom 2, second floor	Clean zone	-	
2022/3/20	Washroom 3, second floor	Clean zone	-	
2022/3/20	Washroom 4, second floor	Clean zone	-	
2022/3/21	Second undressing room 1	Emergency zone	-	
2022/3/21	Second undressing room 2	Emergency zone	-	
2022/3/21	Second undressing room 3	Emergency zone	-	
2022/3/21	Second undressing room 4	Emergency zone	-	

2022/3/21	Second undressing room 5	Emergency zone	-	
2022/3/21	Second undressing room 6	Emergency zone	-	
2022/3/21	Second undressing room 7	Emergency zone	-	
2022/3/21	Second undressing room 8	Emergency zone	-	
2022/3/21	Doctor’s office 1, second floor	Clean zone	-	
2022/3/21	Doctor’s office 2, second floor	Clean zone	-	
2022/3/21	Doctor’s office 3, second floor	Clean zone	-	
2022/3/21	Doctor’s office 4, second floor	Clean zone	-	
2022/3/21	Doctor’s office 5, second floor	Clean zone	-	
2022/3/21	Doctor’s office 6, second floor	Clean zone	-	
2022/3/21	Doctor’s office 7, second floor	Clean zone	-	
2022/3/21	Doctor’s office 8, second floor	Clean zone	-	
2022/3/21	Washroom 1, second floor	Clean zone	-	
2022/3/21	Washroom 2, second floor	Clean zone	-	
2022/3/21	Washroom 3, second floor	Clean zone	-	
2022/3/21	Washroom 4, second floor	Clean zone	-	
2022/3/22	Second undressing room 1	Emergency zone	-	
2022/3/22	Second undressing room 2	Emergency zone	-	
2022/3/22	Second undressing room 3	Emergency zone	-	
2022/3/22	Second undressing room 4	Emergency zone	-	
2022/3/22	Second undressing room 5	Emergency zone	-	
2022/3/22	Second undressing room 6	Emergency zone	-	
2022/3/22	Second undressing room 7	Emergency zone	-	
2022/3/22	Second undressing room 8	Emergency zone	-	
2022/3/22	Doctor’s office 1, third floor, F2-001	Clean zone	-	
2022/3/22	Doctor’s office 2, third floor, F2-020	Clean zone	-	
2022/3/22	Doctor’s office 3, third floor, F2-056	Clean zone	-	
2022/3/22	Doctor’s office 4, third floor, F2-066	Clean zone	-	
2022/3/22	Doctor’s office 5, third floor, F2-100	Clean zone	-	
2022/3/22	Doctor’s office 6, third floor, F2-112	Clean zone	-	
2022/3/22	Doctor’s office 7, third floor, F2-142	Clean zone	-	
2022/3/22	Doctor’s office 8, third floor, F2-163	Clean zone	-	
2022/3/22	Washroom 1, second floor, F2--040	Clean zone	-	
2022/3/22	Washroom 2, second floor, F2-076	Clean zone	-	
2022/3/22	Washroom 3, second floor, F2-122	Clean zone	-	
2022/3/22	Washroom 4, second floor, F2-085	Clean zone	-	
2022/3/23	Second undressing room 1	Emergency zone	-	
2022/3/23	Second undressing room 2	Emergency zone	-	
2022/3/23	Second undressing room 3	Emergency zone	-	
2022/3/23	Second undressing room 4	Emergency zone	-	
2022/3/23	Second undressing room 5	Emergency zone	-	
2022/3/23	Second undressing room 6	Emergency zone	-	
2022/3/23	Second undressing room 7	Emergency zone	-	

2022/3/23	Second undressing room 8	Emergency zone	-	
2022/3/23	Doctor’s office 1, fourth floor, F2-001	Clean zone	-	
2022/3/23	Doctor’s office 1, fourth floor, F2-002	Clean zone	-	
2022/3/23	Doctor’s office 1, fourth floor, F2-003	Clean zone	-	
2022/3/23	Doctor’s office 1, fourth floor, F2-004	Clean zone	-	
2022/3/23	Doctor’s office 1, fourth floor, F2-005	Clean zone	-	
2022/3/23	Doctor’s office 1, fourth floor, F2-006	Clean zone	-	
2022/3/23	Doctor’s office 1, fourth floor, F2-007	Clean zone	-	
2022/3/23	Doctor’s office 1, fourth floor, F2-008	Clean zone	-	
2022/3/23	Washroom 1, fourth floor, F2-040	Clean zone	-	
2022/3/23	Washroom 1, fourth floor, F2-041	Clean zone	-	
2022/3/23	Washroom 1, fourth floor, F2-042	Clean zone	-	
2022/3/23	Washroom 1, fourth floor, F2-043	Clean zone	-	
2022/3/24	Second undressing room 1	Emergency zone	-	
2022/3/24	Second undressing room 2	Emergency zone	-	
2022/3/24	Second undressing room 3	Emergency zone	-	
2022/3/24	Second undressing room 4	Emergency zone	-	
2022/3/24	Second undressing room 5	Emergency zone	-	
2022/3/24	Second undressing room 6	Emergency zone	-	
2022/3/24	Second undressing room 7	Emergency zone	-	
2022/3/24	Second undressing room 8	Emergency zone	-	
2022/3/24	Doctor’s office 1, second floor	Clean zone	-	
2022/3/24	Doctor’s office 2, second floor	Clean zone	-	
2022/3/24	Doctor’s office 3, second floor	Clean zone	-	
2022/3/24	Doctor’s office 4, second floor	Clean zone	-	
2022/3/24	Doctor’s office 5, second floor	Clean zone	-	
2022/3/24	Doctor’s office 6, second floor	Clean zone	-	
2022/3/24	Doctor’s office 7, second floor	Clean zone	-	
2022/3/24	Doctor’s office 8, second floor	Clean zone	-	
2022/3/24	Washroom 1, second floor	Clean zone	-	
2022/3/24	Washroom 2, second floor	Clean zone	-	
2022/3/24	Washroom 3, second floor	Clean zone	-	
2022/3/24	Washroom 4, second floor	Clean zone	-	
2022/3/25	Second undressing room 1	Emergency zone	-	
2022/3/25	Second undressing room 2	Emergency zone	-	
2022/3/25	Second undressing room 3	Emergency zone	-	
2022/3/25	Second undressing room 4	Emergency zone	-	
2022/3/25	Second undressing room 5	Emergency zone	-	
2022/3/25	Second undressing room 6	Emergency zone	-	
2022/3/25	Second undressing room 7	Emergency zone	-	
2022/3/25	Second undressing room 8	Emergency zone	-	
2022/3/25	Electricity distribution room 1, second floor	Clean zone	-	

2022/3/25	Electricity distribution room 2, second floor	Clean zone	-	
2022/3/25	Electricity distribution room 3, second floor	Clean zone	-	
2022/3/25	Lounge 1, second floor, zone II	Clean zone	-	
2022/3/25	Lounge 2, second floor, zone II	Clean zone	-	
2022/3/25	Lounge 3, second floor, zone II	Clean zone	-	
2022/3/25	Lounge 4, second floor, zone II	Clean zone	-	
2022/3/25	Lounge 5, second floor, zone II	Clean zone	-	
2022/3/25	Lounge 6, second floor, zone II	Clean zone	-	
2022/3/25	Lounge 7, second floor, zone II	Clean zone	-	
2022/3/25	Lounge 8, second floor, zone II	Clean zone	-	
2022/3/25	Lounge for cleaner, Second floor	Clean zone	-	
2022/3/26	Electricity distribution room 1, second floor	Clean zone	-	
2022/3/26	Electricity distribution room 2, second floor	Clean zone	-	
2022/3/26	Electricity distribution room 3, second floor	Clean zone	-	
2022/3/26	Lounge 1, second floor, zone II	Clean zone	-	
2022/3/26	Lounge 2, second floor, zone II	Clean zone	-	
2022/3/26	Lounge 3, second floor, zone II	Clean zone	-	
2022/3/26	Lounge 4, second floor, zone II	Clean zone	-	
2022/3/26	Lounge 5, second floor, zone II	Clean zone	-	
2022/3/26	Lounge 6, second floor, zone II	Clean zone	-	
2022/3/26	Lounge 7, second floor, zone II	Clean zone	-	
2022/3/26	Lounge 8, second floor, zone II	Clean zone	-	
2022/3/26	Lounge for cleaner, second floor	Clean zone	-	
2022/3/27	Electricity distribution room 1, second floor	Clean zone	-	
2022/3/27	Electricity distribution room 2, second floor	Clean zone	-	
2022/3/27	Electricity distribution room 3, second floor	Clean zone	-	
2022/3/27	Lounge 1, second floor, zone II	Clean zone	-	
2022/3/27	Lounge 2, second floor, zone II	Clean zone	-	
2022/3/27	Lounge 3, second floor, zone II	Clean zone	-	
2022/3/27	Lounge 4, second floor, zone II	Clean zone	-	
2022/3/27	Lounge 5, second floor, zone II	Clean zone	-	
2022/3/27	Lounge 6, second floor, zone II	Clean zone	-	
2022/3/27	Lounge 7, second floor, zone II	Clean zone	-	
2022/3/27	Lounge 8, second floor, zone II	Clean zone	-	
2022/3/27	Lounge for cleaner, second floor	Clean zone	-	

2022/3/28	Electricity distribution room 1, second floor	Clean zone	-	
2022/3/28	Electricity distribution room 2, second floor	Clean zone	-	
2022/3/28	Electricity distribution room 3, second floor	Clean zone	-	
2022/3/28	Lounge 1, second floor, zone II	Clean zone	-	
2022/3/28	Lounge 2, second floor, zone II	Clean zone	-	
2022/3/28	Lounge 3, second floor, zone II	Clean zone	-	
2022/3/28	Lounge 4, second floor, zone II	Clean zone	-	
2022/3/28	Lounge 5, second floor, zone II	Clean zone	-	
2022/3/28	Lounge 6, second floor, zone II	Clean zone	-	
2022/3/28	Lounge 7, second floor, zone II	Clean zone	-	
2022/3/28	Lounge 8, second floor, zone II	Clean zone	-	
2022/3/28	Lounge for cleaner, second floor	Clean zone	-	
2022/3/29	Electricity distribution room 1, second floor	Clean zone	-	
2022/3/29	Electricity distribution room 2, second floor	Clean zone	-	
2022/3/29	Electricity distribution room 3, second floor	Clean zone	-	
2022/3/29	Lounge 1, second floor, zone II	Clean zone	-	
2022/3/29	Lounge 2, second floor, zone II	Clean zone	-	
2022/3/29	Lounge 3, second floor, zone II	Clean zone	-	
2022/3/29	Lounge 4, second floor, zone II	Clean zone	-	
2022/3/29	Lounge 5, second floor, zone II	Clean zone	-	
2022/3/29	Lounge 6, second floor, zone II	Clean zone	-	
2022/3/29	Lounge 7, second floor, zone II	Clean zone	-	
2022/3/29	Lounge 8, second floor, zone II	Clean zone	-	
2022/3/29	Lounge for cleaner, second floor	Clean zone	-	
2022/3/29	First undressing room 1	Clean zone	-	
2022/3/29	First undressing room 2	Clean zone	-	
2022/3/29	First undressing room 3	Clean zone	-	
2022/3/29	First undressing room 4	Clean zone	-	
2022/3/29	First undressing room 5	Clean zone	-	
2022/3/29	First undressing room 6	Clean zone	-	
2022/3/29	First undressing room 7	Clean zone	-	
2022/3/29	First undressing room 8	Clean zone	-	
2022/3/30	Lounge 1, second floor, zone II	Clean zone	-	
2022/3/30	Lounge 2, second floor, zone II	Clean zone	-	
2022/3/30	Lounge 3, second floor, zone II	Clean zone	-	
2022/3/30	Lounge 4, second floor, zone II	Clean zone	-	
2022/3/30	Lounge 5, second floor, zone II	Clean zone	-	

2022/3/30	Lounge 6, second floor, zone II	Clean zone	-	
2022/3/30	Lounge 7, second floor, zone II	Clean zone	-	
2022/3/30	Lounge 8, second floor, zone II	Clean zone	-	
2022/3/30	Lounge 9, second floor, zone II	Clean zone	-	
2022/3/30	Lounge 10, second floor, zone II	Clean zone	-	
2022/3/30	Infection-control office, zone VIII	Clean zone	-	
2022/3/30	Office, zone V	Clean zone	-	
2022/3/30	First undressing room 1	Clean zone	-	
2022/3/30	First undressing room 2	Clean zone	-	
2022/3/30	First undressing room 3	Clean zone	-	
2022/3/30	First undressing room 4	Clean zone	-	
2022/3/30	First undressing room 5	Clean zone	-	
2022/3/30	First undressing room 6	Clean zone	-	
2022/3/30	First undressing room 7	Clean zone	-	
2022/3/30	First undressing room 8	Clean zone	-	
2022/3/31	Lounge 1, second floor, zone II	Clean zone	-	
2022/3/31	Lounge 2, second floor, zone II	Clean zone	-	
2022/3/31	Lounge 3, second floor, zone II	Clean zone	-	
2022/3/31	Lounge 4, second floor, zone II	Clean zone	-	
2022/3/31	Lounge 5, second floor, zone II	Clean zone	-	
2022/3/31	Lounge 6, second floor, zone II	Clean zone	-	
2022/3/31	Lounge 7, second floor, zone II	Clean zone	-	
2022/3/31	Lounge 8, second floor, zone II	Clean zone	-	
2022/3/31	Lounge 9, second floor, zone II	Clean zone	-	
2022/3/31	Lounge 10, second floor, zone II	Clean zone	-	
2022/3/31	Infection-control office, zone VIII	Clean zone	-	
2022/3/31	Office, zone V	Clean zone	-	
2022/4/1	First undressing room 1	Clean zone	-	
2022/4/1	First undressing room 2	Clean zone	-	
2022/4/1	First undressing room 3	Clean zone	-	
2022/4/1	First undressing room 4	Clean zone	-	
2022/4/1	First undressing room 5	Clean zone	-	
2022/4/1	First undressing room 6	Clean zone	-	
2022/4/2	First undressing room 1	Clean zone	-	
2022/4/2	First undressing room 2	Clean zone	-	
2022/4/2	First undressing room 3	Clean zone	-	
2022/4/2	First undressing room 4	Clean zone	-	
2022/4/2	First undressing room 5	Clean zone	-	
2022/4/2	First undressing room 6	Clean zone	-	

-, test negative result. +, test positive result.

TABLE S3. Sampling sites and test results of viral aerosols in emergency and contaminant zones

Sampling date	Sampling point location	Zone	Results	Ct value
2022/4/1	Outside corridor 1, isolation ward (Door closed)	Contaminant zone	-	
2022/4/1	Outside corridor 2, isolation ward (Door closed)	Contaminant zone	-	
2022/4/1	Outside corridor 3, isolation ward (Door closed)	Contaminant zone	-	
2022/4/1	Outside corridor 4, isolation ward (Door closed)	Contaminant zone	-	
2022/4/2	First undressing room 1	Emergency zone	-	
2022/4/2	First undressing room 2	Emergency zone	-	
2022/4/2	First undressing room 3	Emergency zone	-	
2022/4/2	First undressing room 4	Emergency zone	-	
2022/4/2	First undressing room 5	Emergency zone	-	
2022/4/2	First undressing room 6	Emergency zone	-	
2022/4/2	Outside corridor 1, isolation ward (Door closed)	Contaminant zone	-	
2022/4/2	Outside corridor 2, isolation ward (Door closed)	Contaminant zone	-	
2022/4/2	Outside corridor 3, isolation ward (Door closed)	Contaminant zone	-	
2022/4/2	Outside corridor 4, isolation ward (Door closed)	Contaminant zone	-	
2022/4/2	Empty isolation ward 1	Contaminant zone	-	
2022/4/2	Empty isolation ward 2	Contaminant zone	-	
2022/4/2	Empty isolation ward 3	Contaminant zone	-	
2022/4/2	Empty isolation ward 4	Contaminant zone	-	
2022/4/3	First undressing room 1	Emergency zone	-	
2022/4/3	First undressing room 2	Emergency zone	-	
2022/4/3	First undressing room 3	Emergency zone	-	
2022/4/3	First undressing room 4	Emergency zone	-	
2022/4/3	First undressing room 5	Emergency zone	-	
2022/4/3	First undressing room 6	Emergency zone	-	
2022/4/3	Outside corridor 1, isolation ward (Door closed)	Contaminant zone	-	
2022/4/3	Outside corridor 2, isolation ward (Door closed)	Contaminant zone	-	
2022/4/3	Outside corridor 3, isolation ward (Door closed)	Contaminant zone	-	
2022/4/3	Outside corridor 4, isolation ward (Door closed)	Contaminant zone	-	
2022/4/3	Outside corridor 5, isolation ward (Door closed)	Contaminant zone	-	
2022/4/3	Outside corridor 6, isolation ward (Door closed)	Contaminant zone	-	
2022/4/3	Empty isolation ward 1	Contaminant zone	-	
2022/4/3	Empty isolation ward 2	Contaminant zone	-	
2022/4/3	Empty isolation ward 3	Contaminant zone	-	
2022/4/3	Empty isolation ward 4	Contaminant zone	-	
2022/4/3	Empty isolation ward 5	Contaminant zone	-	
2022/4/3	Empty isolation ward 6	Contaminant zone	-	
2022/4/4	First undressing room 1	Emergency zone	-	
2022/4/4	First undressing room 2	Emergency zone	-	
2022/4/4	First undressing room 3	Emergency zone	-	

2022/4/4	First undressing room 4	Emergency zone	-	
2022/4/4	Outside corridor 1, isolation ward (Door closed)	Contaminant zone	-	
2022/4/4	Outside corridor 2, isolation ward (Door closed)	Contaminant zone	-	
2022/4/4	Outside corridor 3, isolation ward (Door closed)	Contaminant zone	-	
2022/4/4	Outside corridor 4, isolation ward (Door closed)	Contaminant zone	-	
2022/4/4	Outside corridor 5, isolation ward (Door closed)	Contaminant zone	-	
2022/4/4	Outside corridor 6, isolation ward (Door closed)	Contaminant zone	-	
2022/4/4	Outside corridor 7, isolation ward (Door closed)	Contaminant zone	-	
2022/4/4	Outside corridor 8, Isolation ward (Door closed)	Contaminant zone	-	
2022/4/4	Isolation ward 1	Contaminant zone	-	
2022/4/4	Isolation ward 2	Contaminant zone	-	
2022/4/4	Isolation ward 3	Contaminant zone	-	
2022/4/5	First undressing room 1	Emergency zone	-	
2022/4/5	First undressing room 2	Emergency zone	-	
2022/4/5	First undressing room 3	Emergency zone	-	
2022/4/5	First undressing room 4	Emergency zone	-	
2022/4/5	Southern outside corridor 1, isolation ward	Contaminant zone	-	
2022/4/5	Southern outside corridor 2, isolation ward	Contaminant zone	-	
2022/4/5	Southern outside corridor 1, isolation ward	Contaminant zone	+	43.4
2022/4/5	Southern outside corridor 2, isolation ward	Contaminant zone	+	36.1
2022/4/5	Northern outside corridor 3, isolation ward	Contaminant zone	-	
2022/4/5	Northern outside corridor 4, isolation ward	Contaminant zone	-	
2022/4/5	Northern outside corridor 3, isolation ward	Contaminant zone	+	39
2022/4/5	Northern outside corridor 4, isolation ward	Contaminant zone	-	
2022/4/5	Isolation ward 4	Contaminant zone	+	37.5
2022/4/5	Isolation ward 8	Contaminant zone	+	38.1
2022/4/5	Isolation ward 16	Contaminant zone	-	
2022/4/6	First undressing room 1	Emergency zone	-	
2022/4/6	First undressing room 2	Emergency zone	-	
2022/4/6	First undressing room 3	Emergency zone	-	
2022/4/6	First undressing room 4	Emergency zone	-	
2022/4/6	Southern outside corridor 1, isolation ward	Contaminant zone	-	
2022/4/6	Southern outside corridor 2, isolation ward	Contaminant zone	-	
2022/4/6	Southern outside corridor 1, isolation ward	Contaminant zone	-	
2022/4/6	Southern outside corridor 2, isolation ward	Contaminant zone	-	
2022/4/6	Northern outside corridor 3, isolation ward	Contaminant zone	-	
2022/4/6	Northern outside corridor 4, isolation ward	Contaminant zone	-	
2022/4/6	Northern outside corridor 3, isolation ward	Contaminant zone	-	
2022/4/6	Northern outside corridor 4, isolation ward	Contaminant zone	-	
2022/4/6	Isolation ward 4	Contaminant zone	+	41.2
2022/4/6	Isolation ward 8	Contaminant zone	-	
2022/4/7	First undressing room 1	Emergency zone	-	
2022/4/7	First undressing room 2	Emergency zone	-	

2022/4/7	First undressing room 3	Emergency zone	-	
2022/4/7	First undressing room 4	Emergency zone	-	
2022/4/7	Northern outside corridor 1, isolation ward	Contaminant zone	+	38.8
2022/4/7	Northern outside corridor 2, isolation ward	Contaminant zone	-	
2022/4/7	Northern outside corridor 3, isolation ward	Contaminant zone	-	
2022/4/7	Northern outside corridor 4, isolation ward	Contaminant zone	-	
2022/4/7	Northern outside corridor 5, isolation ward	Contaminant zone	-	
2022/4/7	Northern outside corridor 6, isolation ward	Contaminant zone	-	
2022/4/7	Northern outside corridor 7, isolation ward	Contaminant zone	-	
2022/4/7	Northern outside corridor 8, isolation ward	Contaminant zone	-	
2022/4/7	Isolation ward 4	Contaminant zone	-	
2022/4/7	Isolation ward 8	Contaminant zone	-	
2022/4/8	First undressing room 1	Emergency zone	-	
2022/4/8	First undressing room 2	Emergency zone	-	
2022/4/8	First undressing room 3	Emergency zone	-	
2022/4/8	First undressing room 4	Emergency zone	-	
2022/4/8	Northern outside corridor 1, isolation ward	Contaminant zone	-	
2022/4/8	Northern outside corridor 2, isolation ward	Contaminant zone	-	
2022/4/8	Northern outside corridor 3, isolation ward	Contaminant zone	-	
2022/4/8	Northern outside corridor 4, isolation ward	Contaminant zone	+	37.8
2022/4/8	Northern outside corridor 5, isolation ward	Contaminant zone	-	
2022/4/8	Northern outside corridor 6, isolation ward	Contaminant zone	-	
2022/4/8	Northern outside corridor 7, isolation ward	Contaminant zone	-	
2022/4/8	Northern outside corridor 8, isolation ward	Contaminant zone	-	
2022/4/9	First undressing room 1	Emergency zone	-	
2022/4/9	First undressing room 2	Emergency zone	-	
2022/4/9	First undressing room 3	Emergency zone	-	
2022/4/9	First undressing room 4	Emergency zone	-	
2022/4/9	Northern outside corridor 1, isolation ward	Contaminant zone	-	
2022/4/9	Northern outside corridor 2, isolation ward	Contaminant zone	-	
2022/4/9	Northern outside corridor 3, isolation ward	Contaminant zone	-	
2022/4/9	Northern outside corridor 4, isolation ward	Contaminant zone	-	
2022/4/9	Northern outside corridor 5, isolation ward	Contaminant zone	-	
2022/4/9	Northern outside corridor 6, isolation ward	Contaminant zone	-	
2022/4/9	Northern outside corridor 7, isolation ward	Contaminant zone	-	
2022/4/9	Northern outside corridor 8, isolation ward	Contaminant zone	-	

-, test negative result. +, test positive result.

TABLE S4. Test results of swab samples collected from COVID-19 patients in corresponding wards

Sampling data	Ward	Bed	Admission data	Results	Ct value	Days from admission
2022/4/5	E6-1	01	2022/3/16	-	-	20 days
2022/4/5	E6-2	04	2022/4/8	/	/	/
2022/4/5	E6-2	03	2022/4/8	/	/	/
2022/4/5	E6-3	02	2022/3/16	#	#	20 days
2022/4/5	E6-3	05	2022/4/3	#	#	2 days
2022/4/5	E6-4	08	2022/4/3	#	#	2 days
2022/4/5	E6-5	09	2022/3/25	+	Ct=37.32	11 days
2022/4/5	E6-5	10	2022/4/4	#	#	1 days
2022/4/5	E6-6	11	2022/4/3	-	-	3 days
2022/4/5	E6-6	21	2022/4/7	/	/	/
2022/4/5	E6-8	16	2022/4/1	#	#	4 days
2022/4/5	E6-9	17	2022/4/7	/	/	/
2022/4/5	E6-9	18	2022/4/8	/	/	/
2022/4/5	E6-11	21	2022/4/6	/	/	/
2022/4/5	E6-12	23	2022/4/6	/	/	/
2022/4/5	E6-12	24	2022/4/4	+	Ct=23.21	1 days
2022/4/5	E6-13	26	2022/4/4	+	Ct=22.15	1 days
2022/4/5	E6-14	28	2022/4/5	+	Ct=22.7	0 days
2022/4/5	E6-14	27	2022/4/5	#	#	0 days
2022/4/5	E6-15	03	2022/4/1	#	#	4 days
2022/4/5	E6-16	32	2022/3/30	+	Ct=30.21	6 days
2022/4/5	E6-17	33	2022/4/1	+	Ct=36.12	4 days
2022/4/5	E6-18	36	2022/3/17	+	Ct=36.52	19 days
2022/4/6	E6-1	01	2022/3/16	-	-	21 days
2022/4/6	E6-2	04	2022/4/8	/	/	/
2022/4/6	E6-2	03	2022/4/8	/	/	/
2022/4/6	E6-3	02	2022/3/16	#	#	21 days
2022/4/6	E6-3	05	2022/4/3	#	#	3 days
2022/4/6	E6-4	08	2022/4/3	#	#	3 days
2022/4/6	E6-5	09	2022/3/25	+	Ct=38.56	12 days
2022/4/6	E6-5	10	2022/4/4	+	Ct=21.49	2 days
2022/4/6	E6-6	11	2022/4/3	#	#	4 days
2022/4/6	E6-6	21	2022/4/7	/	/	/
2022/4/6	E6-8	16	2022/4/1	#	#	5 days
2022/4/6	E6-9	17	2022/4/7	/	/	/
2022/4/6	E6-9	18	2022/4/8	/	/	/
2022/4/6	E6-11	21	2022/4/6	+	Ct=20.15	0 days
2022/4/6	E6-12	23	2022/4/6	+	Ct=30.57	0 days
2022/4/6	E6-12	24	2022/4/4	+	Ct=24.11	2 days

2022/4/6	E6-13	26	2022/4/4	+	Ct=25.46	2 days
2022/4/6	E6-14	28	2022/4/5	+	Ct=21.59	1 days
2022/4/6	E6-14	27	2022/4/5	+	Ct=24.85	1 days
2022/4/6	E6-15	03	2022/4/1	#	#	5 days
2022/4/6	E6-16	32	2022/3/30	#	#	7 days
2022/4/6	E6-17	33	2022/4/1	#	#	5 days
2022/4/6	E6-18	36	2022/3/17	+	Ct=34.12	20 days
2022/4/7	E6-11	21	2022/4/6	+	Ct=22.06	1 days
2022/4/7	E6-12	23	2022/4/6	#	#	1 days
2022/4/7	E6-12	24	2022/4/4	+	Ct=30.8	3 days
2022/4/7	E6-13	26	2022/4/4	+	Ct=27.05	3 days
2022/4/7	E6-14	28	2022/4/5	#	#	2 days
2022/4/7	E6-14	27	2022/4/5	#	#	2 days
2022/4/7	E6-15	03	2022/4/1	#	#	6 days
2022/4/7	E6-16	32	2022/3/30	#	#	8 days
2022/4/7	E6-17	33	2022/4/1	-	-	6 days
2022/4/7	E6-18	36	2022/3/17	#	#	21 days
2022/4/8	E6-11	21	2022/4/6	+	Ct=24.95	2 days
2022/4/8	E6-12	23	2022/4/6	#	#	2 days
2022/4/8	E6-12	24	2022/4/4	+	Ct=26.66	4 days
2022/4/8	E6-13	26	2022/4/4	+	Ct=31.55	4 days
2022/4/8	E6-14	28	2022/4/5	#	#	3 days
2022/4/8	E6-14	27	2022/4/5	#	#	3 days
2022/4/8	E6-15	03	2022/4/1	+	Ct=32.65	7 days
2022/4/8	E6-16	32	2022/3/30	+	Ct=35.92	9 days
2022/4/8	E6-17	33	2022/4/1	-	-	7 days
2022/4/8	E6-18	36	2022/3/17	+	Ct=26.54	22 days
2022/4/9	E6-11	21	2022/4/6	+	Ct=26.65	3 days
2022/4/9	E6-12	23	2022/4/6	#	#	3 days
2022/4/9	E6-12	24	2022/4/4	-	-	5 days
2022/4/9	E6-13	26	2022/4/4	+	Ct=38.27	5 days
2022/4/9	E6-14	28	2022/4/5	+	Ct=28.57	4 days
2022/4/9	E6-14	27	2022/4/5	+	Ct=27.05	4 days
2022/4/9	E6-15	03	2022/4/1	#	#	8 days
2022/4/9	E6-16	32	2022/3/30	+	Ct=38.58	10 days
2022/4/9	E6-17	33	2022/4/1	#	#	8 days
2022/4/9	E6-18	36	2022/3/17	+	Ct=37.97	23 days

#, without the data of nucleic acid testing yet with COVID-19 patient in the ward. /, without COVID-19 patients. -, test negative result. +, test positive result.
